# A registry study of relapsed or refractory multiple myeloma pre‐exposed to three or more prior therapies including a proteasome inhibitor, an immunomodulatory agent and CD38‐targeted monoclonal antibody therapy in England

**DOI:** 10.1002/jha2.214

**Published:** 2021-06-10

**Authors:** Ahmed Elsada, Amy Zalin‐Miller, Craig Knott, Leonidas Caravotas

**Affiliations:** ^1^ Janssen UK High Wycombe UK; ^2^ Health Data Insight CIC Cambridge UK; ^3^ National Disease Registration Service Public Health England London UK

**Keywords:** overall survival, real‐world data, relapsed or refractory multiple myeloma, salvage therapy, triple‐class exposed

## Abstract

Some patients with multiple myeloma are receiving treatment in clinical practice in England after prior exposure to a proteasome inhibitor, an immunomodulatory agent, and an anti‐CD38 monoclonal antibody. We investigated the characteristics of these patients, their outcomes, and the salvage therapies they received using the national cancer registry for England and linked healthcare data. After a median follow‐up time of 6.4 months from *T*_0_, median overall survival and time to next treatment were 8.2 and 5.3 months, respectively. This real‐world data provide useful clinical insight into a little‐studied patient population and highlight the poor outcomes in the UK setting.

## 1 INTRODUCTION

The advent of anti‐CD38 monoclonal antibodies (MoABs) has led a new relapsed or refractory multiple myeloma (RRMM) population to emerge in recent years encompassing patients pre‐exposed to at least one proteasome inhibitors (PI), one immunomodulatory agent (IMiD), and an anti‐CD38 MoAB, separately or in combination (hereafter referred to as ‘triple‐class exposed’). In England, National Health Service (NHS) patients pre‐treated with a PI and IMiD will generally become triple‐class exposed upon receiving anti‐CD38 MoAB‐based therapy after 1 [1] or, more commonly, 3 [2, 3] prior lines of treatment (LOTs) since second‐line CD38‐targeted therapy has become available relatively recently [1].

Few options are available for the treatment of triple‐class‐exposed RRMM [4]; these include conventional chemotherapy, salvage autologous stem cell transplantation, and/or re‐treatment with previously received regimens [4]. Recently, the first treatment post CD38‐targeted therapy – belantamab mafodotin – was approved in Europe, although this is not currently available on the NHS.

There are very limited data on triple‐class‐exposed RRMM, but what data there are point toward the poor prognosis in US patients [5, 6] and more so in UK patients [7, 8]. To this end, we set up a retrospective cohort study to describe the clinical picture of heavily pre‐treated (after three prior LOTs), triple‐class‐exposed RRMM in England.

## 2 METHODS

The study utilised several linked datasets available through the National Cancer Registration and Analysis Service (NCRAS) at Public Health England (PHE) including the National Cancer Registration Dataset (NCRD) [9], Hospital Episode Statistics database (HES) [10], and the Systemic Anti‐Cancer Therapy dataset (SACT) [11].

The eligible population included patients with a primary MM diagnosis (International Classification of Disease of Oncology morphology code 9732) between 01 January 2013 and 31 December 2018 in England and aged ≥18 years at diagnosis. They must have initiated a new line of systemic anti‐cancer therapy (‘index LOT’) after prior receipt of three or more LOTs including a PI, IMIiD, and anti‐CD38 MoAB. The index LOT must have contained at least one specific MM regimen of interest (Supporting Information Materials Section S1). Patients were excluded if their MM diagnosis was via death certificate only, or if there was no linkage to SACT for an International Classification of Diseases (tenth revision, ICD‐10) C90 tumour, where treatment was after or up to 1 month before the first cohort‐relevant diagnosis. Patients were followed from *T*_0_, defined as the start of the index LOT, to the earliest of death, embarkation (relocation outside England) or 31 December 2019.

As LOTs are not reported in SACT, they were derived using a regimen‐based algorithm (Supporting Information Materials Section S2).

To identify patients with refractory disease per International Myeloma Working Group (IMWG) [12], we utilised the duration of the treatment‐free interval between LOT, as information reflecting the formal IMWG criteria is not readily available in the SACT dataset. In consultation with clinicians, refractory disease to a prior therapy was defined whenever patients started the next LOT ≤60 days after ending the preceding LOT, assuming that in clinical practice, patients whose disease becomes refractory to a LOT are likely to move to the next LOT within 2 months to prevent organ damage. The 60‐day treatment‐free interval was also utilised in a validated algorithm to derive refractory status [13]. A sensitivity analysis was performed by changing the gap to ≤30 and ≤90 days. Patients could be refractory to multiple lines of prior therapy.

Median follow‐up time from *T*_0_ was calculated using the Kaplan–Meier reverse censoring method. Overall survival (OS) and time to next treatment (TTNT) were calculated using the Kaplan–Meier estimator. OS failure was defined as death from any cause between *T*_0_ and the end of follow‐up. TTNT failure was the earliest of either a change in LOT or death within the study period. Patients were censored on 31 December 2019 if alive at the end of the study period, or else on the date of embarkation.

## 3 RESULTS

The cohort had 366 patients (Supporting Information Materials Section S3). Median follow‐up time was 6.4 months from *T*_0_ (95% CI 5.9–7.3).

For over 65% of the patients, the index LOT consisted of a pomalidomide‐based regimen (Table 1). The next most used therapy, albeit after a large gap, is panobinostat plus bortezomib, with around 10% of patients receiving it as their index LOT.

**TABLE 1:** Regimens used in index lines of treatment (LOTs)

Index regimen (N, %)		
Pomalidomide	239	65.3%
Panobinostat + bortezomib	42	11.5%
Lenalidomide	13	3.6%
Pomalidomide + bortezomib	10	2.7%
Bendamustine	9	2.5%
DT‐PACE	9	2.5%
Bendamustine + thalidomide	8	2.2%
Cyclophosphamide	8	2.2%
Other	28	7.7%

Abbreviation: DT‐PACE, dexamethasone + thalidomide + cisplatin + doxorubicin + cyclophosphamide + etoposide.

Utilising the proxy definition of refractoriness with a gap ≤60 days between LOTs, 63.1% of patients were lenalidomide‐refractory (Table 2). This was compared to a similar UK population for external validity which showed a 61% lenalidomide‐refractoriness^7^, thereby confirming the gap was suitable.

Demographics, baseline characteristics, and prior therapy are described in Table 2.

**TABLE 2:** Demographics, baseline characteristics, and prior therapy

Variable		Value
Overall cohort, N (%)^a^		366 (100)
Demographics and baseline characteristics		
Age at *T*_0_, years^b^		
	Mean (standard deviation)	70.5 (9.3)
	Median	71.7
	Q1–Q3	64.5–77.2
	Minimum–maximum	42.8–91.7
Age category at *T*_0_, N (%)^b^		
	<65	97 (25.5)
	≥65	272 (74.3)
Gender, N (%)		
	Male	215 (58.7)
	Female	151 (41.3)
Ethnicity, N (%)		
	White	326 (89.1)
	Asian	9 (2.5)
	Black	18 (4.9)
	Other	7 (1.9)
	Unknown	6 (1.6)
Performance status at *T*_0_, ECOG, N (%)^b^		
	0	64 (17.5)
	1	130 (35.5)
	2	102 (27.9)
	3	>10 (–)
	4	<6 (–)
	Unknown	53 (14.5)
Stage at diagnosis, N (%)^d^		
	I	30 (8.2)
	II	27 (7.4)
	III	58 (15.8)
	Unknown	251 (68.6)
Time since diagnosis until start of the index LOT, months		
	Mean (standard deviation)	44.7 (16.8)
	Median	44.0
	Q1–Q3	32.5–55.9
	Minimum–maximum	9.2–81.2
Prior therapy		
Count of prior lines of therapy before index LOT		
	Mean (standard deviation)	3.8 (0.7)
	Median	4
	Q1–Q3	3–4
	Minimum–maximum	3–7
Grouped count of prior lines of therapy before index LOT, N (%)		
	3	132 (36.1)
	4	184 (50.3)
	5	42 (11.5)
	>5	8 (2.2)
Penta exposure, N (%)^e^		
	Yes	83 (22.7)
	No	283 (77.3)
Prior autologous stem cell transplant, N (%)^f^		
	Yes	114 (31.1)
	No	252 (68.9)
PI and IMiD refractoriness, N (%)^g^		
	Yes	178 (48.6)
	No	188 (51.4)
Triple‐class refractoriness, N (%)^h^		
	Yes	157 (42.9)
	No	209 (57.1)
Penta refractoriness, N (%)^i^		
	Yes	12 (3.3)
	No	354 (96.7)
Lenalidomide refractoriness, N (%)^j^		
	Yes	231 (63.1)
	No	135 (36.9)

*Note*: Suppression: values <6 have been replaced with <6 to maintain patient confidentiality, and secondary suppression, with values rounded to nearest 10, applied where small counts could be backwards calculated.

Abbreviations: ECOG, Eastern Cooperative Oncology Group; IMiD, immunomodulatory imide drug.; PI, proteasome inhibitor; Q, quartile.

^a^ Patients with at least one primary cohort‐relevant cancer diagnosis dated between 1 January, 2013 and 31 December 2018, with follow‐up to 31 December 2019 inclusively. Where multiple primary cohort‐relevant cancer diagnoses were documented, the characteristics of the first diagnosis are reported.

^b^ *T*_0_ is the start of the index LOT.

^c^ End of follow‐up is the earliest of death, embarkation or the end of the observation period (31 December 2019).

^d^ For multiple myeloma, the ISS staging system is used.

^e^ Penta exposure indicates whether prior lines of therapy comprised at least two distinct PIs, at least two distinct IMiDs, and one anti‐CD38 monoclonal antibody in any combination.

^f^ Whether at least 1 autologous stem cell transplant was documented during a HES inpatient episode dated between the start of first‐line therapy and the end of the final line prior to *T*_0_. Stem cell transplants are defined using Office of Population Censuses and Surveys Classification of Surgical Operations and Procedures (4th revision) (OPCS‐4) code X334.

^g^ Patients who were refractory to prior lines of therapy containing a PI and an IMiD, separately or in combination. Patients were counted if they were or were not also refractory to an anti‐CD38 monoclonal antibody.

^h^ Patients who were refractory to prior lines of therapy containing a PI, an IMiD, and an anti‐CD38 monoclonal antibody.

^i^ Patients who were refractory to prior lines of therapy containing at least two PIs administered separately with or without other drugs, at least two IMiDs administered separately with or without other drugs, and an anti‐CD38 monoclonal antibody administered with or without other drugs. Patients were not counted if they were refractory to just one PI or IMiD.

^j^ Patients who were refractory to one or more prior lines of therapy containing lenalidomide with or without other drugs.

**FIGURE 1:**
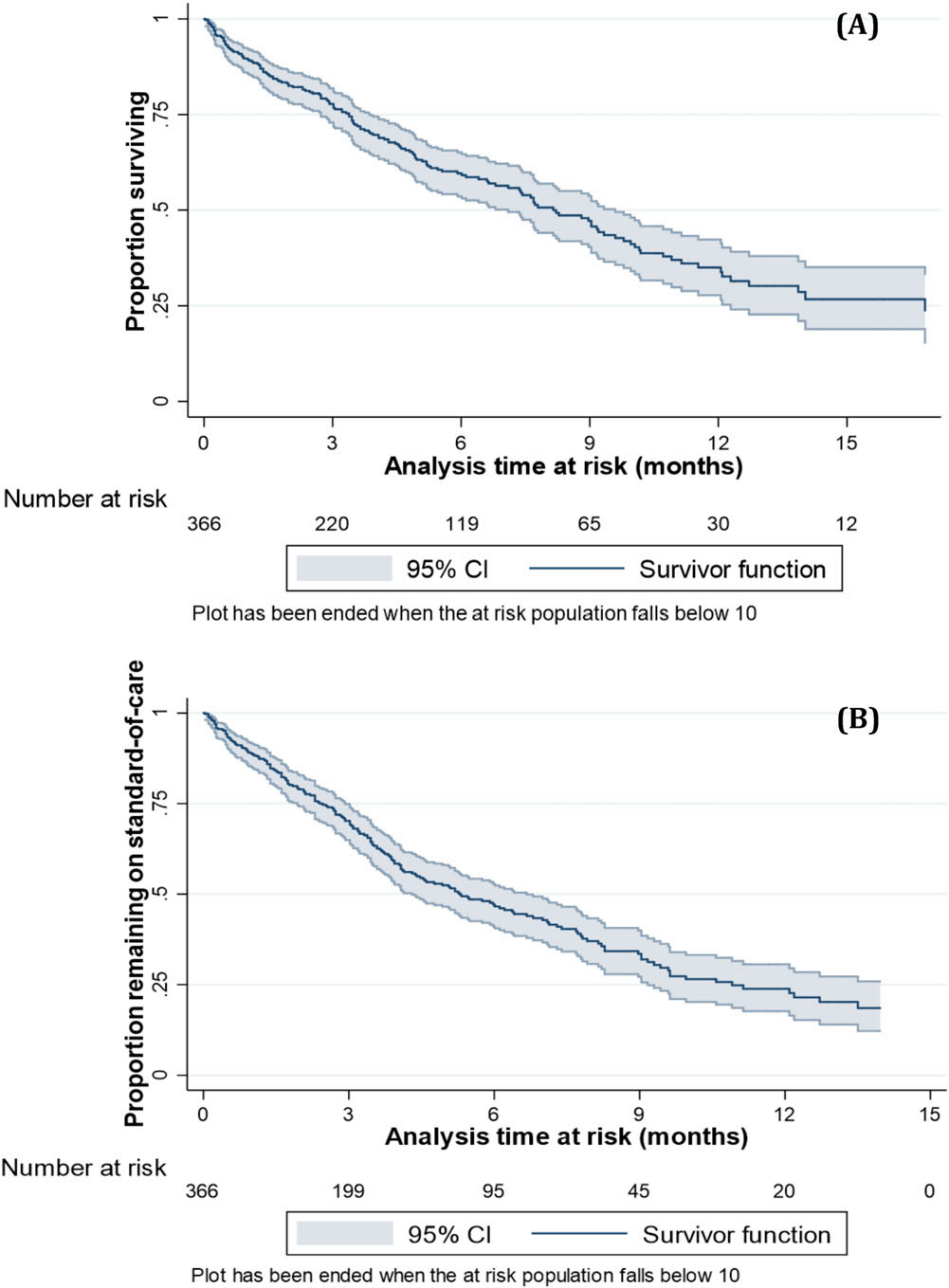
(A) Kaplan–Meier survival curve for overall survival. Failure was defined as death from any cause between *T*_0_ and end of the study period (31 December 2019). (B) Kaplan–Meier survival curve for time to next treatment. Failure was the earliest of either a change in line of treatment (LOT) or death within the study period

Of the 366 patients, 167 (46%) died (Figure 1A) and 199 (54%) died or changed treatment (Figure 1B). Median OS was 8.2 months from *T*_0_ (95% CI 7.1–9.6). At 6 and 12 months, the survivor function was 0.60 (95% CI 0.54–0.65) and 0.35 (95% CI 0.28–0.42), respectively. Median TTNT was 5.29 months (95% CI 4.30–6.67), with a survivor function at 6 months of 0.47 (95% CI 0.41–0.53).

Of the 366 patients in the cohort, 69 (18.9%) moved onto subsequent salvage therapy within the study period. They received 82 distinct regimens at any LOT after the index LOT, mainly a histone deacetylase inhibitor with PI (23 counts) or an IMiD alone (21 counts) or with chemotherapy (14 counts).

## 4 DISCUSSION

To our knowledge, this study is the first to investigate triple‐class‐exposed RRMM in England. It utilises information from a comprehensive registry covering all cancer diagnoses in England, thus providing highly representative and detailed clinical information linked across a wealth of secondary care data. Additionally, the timeliness of these data allowed us to capture recent changes to standard‐of‐care treatment, notably, the use of anti‐CD38 MoABs. The study is limited by the use of an algorithm to derive LOTs, which carries a small risk of having misclassified some patients, by the reliance on a proxy definition of disease refractoriness, and by the scope of the registry which did not allow additional variables of clinical value such as disease cytogenetics to be captured.

With a median OS from *T*_0_ of 8.2 months – reflecting shorter survival than even in triple‐class‐refractory patients in the United States [5, 6] – there is substantial unmet need associated with triple‐class‐exposed RRMM where quality of life worsens, disease burden increases, organ function continues to be impaired, and long‐term exposure to successive treatments requires strategies to manage cumulative toxicities [14]. Median TTNT, reflecting several factors including end‐organ damage, patient preference, and the nature of relapse (indolent vs. proliferative) [13], is also considerably short at 5.3 months. Furthermore, upon disease progression, patients are left with few, suboptimal treatment options [4], highlighting the need to recruit into clinical trials for investigational targets.

In our study, only 1295 (4.6%) of all MM patients in England diagnosed between 2013 and 2018 were triple‐class exposed. This will increase in the future as CD‐38 targeted therapy is now routinely available and moving up the treatment pathway. In fact, regimens combining all three drug classes are being developed for first‐line treatment [15], so some patients may soon become triple‐class exposed on their first LOT, highlighting the need for novel treatment modalities as the disease becomes increasingly difficult to treat in earlier settings.

## AUTHOR CONTRIBUTIONS

All authors made substantial contributions to research design, or the acquisition, analysis or interpretation of data. Ahmed Elsada and Craig Knott designed the research study and wrote the study protocol, with medical input from Leonidas Caravotas. Amy Zalin‐Miller performed the analysis. Ahmed Elsada and Amy Zalin‐Miller wrote the first draft of the manuscript. Leonidas Caravotas contributed to the writing of the manuscript. All authors reviewed and approved the final manuscript.

## CONFLICT OF INTEREST

This study was supported by Janssen UK and carried out by Health Data Insight CIC in partnership with Public Health England. Ahmed Elsada and Leonidas Caravotas are employees of Janssen UK. Amy Zalin‐Miller and Craig Knott are employees of Health Data Insight CIC working in partnership with Public Health England; the latter runs the dataset used for this study.

## Supporting information

Supporting Information

## Data Availability

The data that support the findings of this study are available from the National Disease Registration Service, Public Health England. Restrictions apply to the availability of these data, which were used under license for this study. Data are available from the authors with the permission of the National Disease Registration Service, Public Health England.
