# Supplementary material for: A registry study of relapsed or refractory multiple myeloma pre‐exposed to three or more prior therapies including a proteasome inhibitor, an immunomodulatory agent and CD38‐targeted monoclonal antibody therapy in England
Source: EJHaem. 2021 Jun 10;2(3):493–7. doi: 10.1002/jha2.214 (PMC9175886; doi:10.1002/jha2.214)
Supplement: Supplementary file 1 — Supporting Information [file JHA2-2-493-s001.docx]

**Supplementary Materials Section 1:**

Table 1. Multiple myeloma regimens and drug classes of interest for the index LOT.

| **Regimen** | **Drug Class** |
| --- | --- |
| Bendamustine | Chemotherapy |
| Bortezomib | PI |
| Carfilzomib | PI |
| Cyclophosphamide | Chemotherapy |
| Daratumumab | MoAB (anti-CD38) |
| Doxorubicin | Chemotherapy |
| Elotuzumab | MoAB |
| Ixazomib | PI |
| Lenalidomide | IMiD |
| Melphalan | Chemotherapy |
| Panobinostat | HDAC inhibitor |
| Pomalidomide | IMiD |
| Thalidomide | IMiD |
| Abbreviations: PI, proteasome inhibitor; MoAB, monoclonal antibodies IMiD, immunomodulatory imide drug; HDAC, histone deacetylase. | |

**Supplementary Materials Section 2:**

***Lines of systemic anti-cancer therapy***

Regimens were extracted from SACT if associated with an ICD-10 diagnosis code C90 and dated between up to one month prior to the first cohort-relevant diagnosis as documented in the NCRD and the end of follow-up.

Supportive therapy and trial regimens were excluded. Regimens were restricted to those that included at least one of the drugs in Supplementary Materials Section 1, specifically indicated for MM in England. Steroids of interest (dexamethasone and prednisolone) were extracted from administration-level data where present and added to regimen labels.

Melphalan and cyclophosphamide monotherapy regimens (administered without steroids) were excluded as they are typically used as conditioning regimens prior to transplant and therefore should not be considered for the derivation of LOTs.

For the purposes of deriving LOTs, induction therapy, stem cell transplant (autologous or allogeneic) and consolidation/maintenance therapy were to be counted as one line.

Change in a LOT was defined when:

- All drugs changed between consecutive regimens; or,
- The composition of drug classes changed between consecutive regimens; or,
- The composition of drugs changed between consecutive regimens (but the drug classes remained unchanged); or,
- Any change in the composition of drugs between consecutive regimens where the treatment-free interval was >60 days. The treatment-free interval was the period between the end of a current, and start of a subsequent, regimen.

Exceptions to the LOT rules above were as follows:

- The addition or removal of steroids between consecutive regimens were not considered when applying the change in treatment algorithm.
- Reductions from ‘bortezomib + daratumumab + dexamethasone’ to ‘daratumumab’ with or without steroids was handled as movement to maintenance therapy in instances where the gap between regimens was ≤60 days (gaps larger than this were considered unlikely to be attributable to a move to maintenance therapy). This shift to maintenance therapy was not considered as an initiation of a new LOT.
- LOTs for patients who received a stem cell transplant were derived in the same manner except the treatment-free interval for any change in the composition of drugs (e.g., those used for induction therapy) was extended to >100 days for determining a change from first-line to second-line therapy. From second line the interval reverted to >60 days. This 100-day gap between first- and second-line treatment allowed for induction therapy, stem cell transplant and consolidation/maintenance therapy to be counted as one line.

Regimen duration spanned the earliest known cycle or administration start date and last known cycle or administration date for the regimen. Where regimens contained a single cycle or administration start date (regimen duration 0 days), the duration was imputed as the ‘usual’ cycle duration for that regimen, which was defined in collaboration with a pharmacist.

Myeloma treatment differs according to eligibility for stem cell transplantation. Transplant eligibility was derived according to whether a patient received an autologous or allogenic stem cell transplant from one month prior to diagnosis, to the end of follow-up. A stem cell transplantation flag was created by searching for Office of Population Censuses and Surveys Classification of Surgical Operations and Procedures (4^th^ revision) procedure codes X33.4 or X33.6 in HES inpatient and outpatient procedures data. This was later used in the application of the LOT algorithm whereby the treatment-free interval for any change in the composition of drugs was extended to >100 days for a change from first-line to second-line therapy for patients who received a stem cell transplant, as per the exceptions to the LOT rules stated above.

**Supplementary Materials Section 3:**


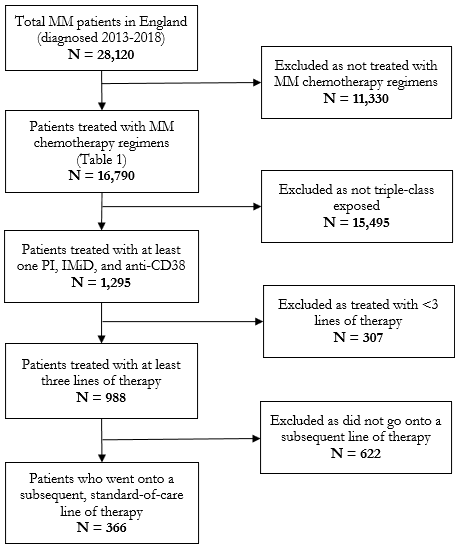


**Figure 2.** Flow chart of patients included in the study.
